# Temperature and heat‐load exposure effects on preterm births: Insights from a population‐based study using distributed lag non‐linear models

**DOI:** 10.1002/ijgo.71112

**Published:** 2026-06-05

**Authors:** Bernard Barzilay, Ibrahim Abu‐Kishk, Haim Bibi, Dan Waisman, Sharon Daniel

**Affiliations:** ^1^ Mayanei Hayeshua Medical Center Bnei Brak Israel; ^2^ Sackler Faculty of Medicine Tel Aviv University Tel Aviv Israel; ^3^ Pediatric Intensive Care Unit Shamir Medical Center (Assaf Harofeh) Zerifin Israel; ^4^ Adelson School of Medicine Ariel University Ariel Israel; ^5^ Carmel Medical Center Haifa Israel; ^6^ The Rappaport School of Medicine, Technion Israel Institute of Technology Haifa Israel; ^7^ Faculty of Health Sciences, Department of Public Health Ben‐Gurion University of the Negev Beer Sheva Israel; ^8^ Faculty of Health Sciences, Department of Pediatrics Ben‐Gurion University of the Negev Beer Sheva Israel; ^9^ Clalit Health Services (Southern District) Beer Sheva Israel

**Keywords:** distributed lag non‐linear models, global warming, heat load, premature birth, preterm delivery

## Abstract

**Objective:**

Preterm birth presents a substantial health, psychological, and economic burden worldwide. Environmental factors, including temperature fluctuations, are known to influence the risk of preterm birth. This study aimed to investigate the association between high temperatures and heat load and preterm births.

**Methods:**

Using a time‐series analysis, we examined data from all births in five Israeli districts between 2010–2016. Meteorological indices were computed for each district. Distributed lag non‐linear models (DLNM) incorporating Poisson regression were employed to investigate the associations between these meteorological indices and the daily number of preterm births across various time lags, assessing both short‐ and long‐term associations, adjusting for seasonality, holidays and the general trend.

**Results:**

During the study period, 1 216 040 newborns were delivered, of which 89 544 (7.36%) were preterm (<37 weeks) and 11 154 (0.91%) early preterm (<32 weeks). Significant non‐linear associations were found between temperature, heat load, and daily preterm births. Acute exposure to a 1°C increase in mean daily temperature 14 days before delivery was associated with a higher risk of preterm birth (relative risk [RR] = 1.015; 95% confidence interval [CI]: 1.010–1.019). Similarly, acute exposure to a 1°C increase in maximal heat load 14 days before delivery was associated with elevated risk (RR = 1.018; 95% CI: 1.011–1.026). Cumulative effects over 14 days were substantial: a 1°C rise in mean daily temperature was linked to a 16% increase in risk (RR = 1.160; 95% CI: 1.145–1.176), while a 1°C increase in maximal heat load was associated with a 14% increase (RR = 1.139; 95% CI: 1.125–1.153). Similar associations were observed for heat load, and for early preterm and very low birth weight births; however, these subgroup analyses were based on fewer events and had wider CIs.

**Conclusion:**

High temperatures and heat load increase the risk of preterm birth over both short‐ and long‐terms. In the context of global warming, these findings may warrant consideration of climate‐related risks in maternal health planning and to develop preventive strategies against heat‐related pregnancy complications.

## 1 INTRODUCTION

Preterm birth constitutes a significant health, psychological, and economic challenge,^1^, ^2^, ^3^, ^4^, ^5^, ^6^, ^7^, ^8^ driving various prevention initiatives. While some factors are preventable,^9^, ^10^ others stem from environmental causes. Pregnant women, undergoing hormone‐related physiological changes, cardiorespiratory adaptation, and fetal size variations, are highly sensitive to weather fluctuations and environmental exposures, affecting their immunological status.^11^, ^12^, ^13^ Given the global rise in temperatures, understanding its potential implications on maternal and fetal health, particularly concerning preterm deliveries, is essential. Studies exploring the relationship between pregnant women's exposure to high environmental temperatures and preterm birth have shown varied results across different countries and settings, indicating associations with preterm delivery^14^, ^15^, ^16^, ^17^, ^18^, ^19^ stillbirth, and low birth weight in both short‐term and chronic exposures. However, many prior distributed lag non‐linear (DLNM) studies have been conducted in high‐income temperate regions and often lacked adjustment for humidity.

Israel's diverse climatic conditions,^20^ influenced by its varied topography, offer a unique opportunity to investigate this relationship. With distinct weather patterns characterized by pronounced seasonal variations, especially noticeable in desert regions, Israel provides a rich environment for studying the impact of temperature fluctuations on preterm deliveries. Therefore, the aim of the present nationwide, population‐based study was to assess the risk of preterm deliveries following exposure to high temperatures and heat load, employing a complex DLNM time‐series model,^21^, ^22^ which allows for the examination of short‐ and long‐term associations of both acute and chronic exposures, providing a comprehensive understanding of the temporal dynamics between environmental factors and preterm deliveries.

## 2 MATERIALS AND METHODS

We conducted a time‐series analysis of all births delivered in five different districts of Israel.

### 2.1 Births data

Data concerning the daily number of births delivered in the different Israeli districts (North, Haifa, Tel‐Aviv, Jerusalem, and the Negev) were retrieved from the Israeli Bureau of Statistics. Daily birth numbers are reported to the Bureau of Statistics by all Israeli hospitals, ensuring a thorough representation of daily births nationwide. The data contained the daily total number of births, preterm births (<37th gestational week), early preterm births (<32nd gestational week), and the daily number of births that resulted in very low birth weight (<1500 g) infants in each district.

### 2.2 Meteorological data

Meteorological data, including temperature and heat load, were obtained from different measuring stations throughout Israel using the Israeli Meteorological Services (IMS) databases.^23^ The database contain meteorological data collected at intervals ranging from 5 min to 1 h at 83 stations situated throughout Israel. The data were gathered from stations situated at the geographical center of each district (Appendix S1). To calculate different meteorological indices for each of the five districts, we used data from stations located at the center of each district (Figure 1). For each district, we calculated the daily mean and maximum temperature, as well as the heat load. Data completeness was very high, with measurements recorded at high temporal resolution and no days in which exposure indices could not be calculated.

**FIGURE 1:**
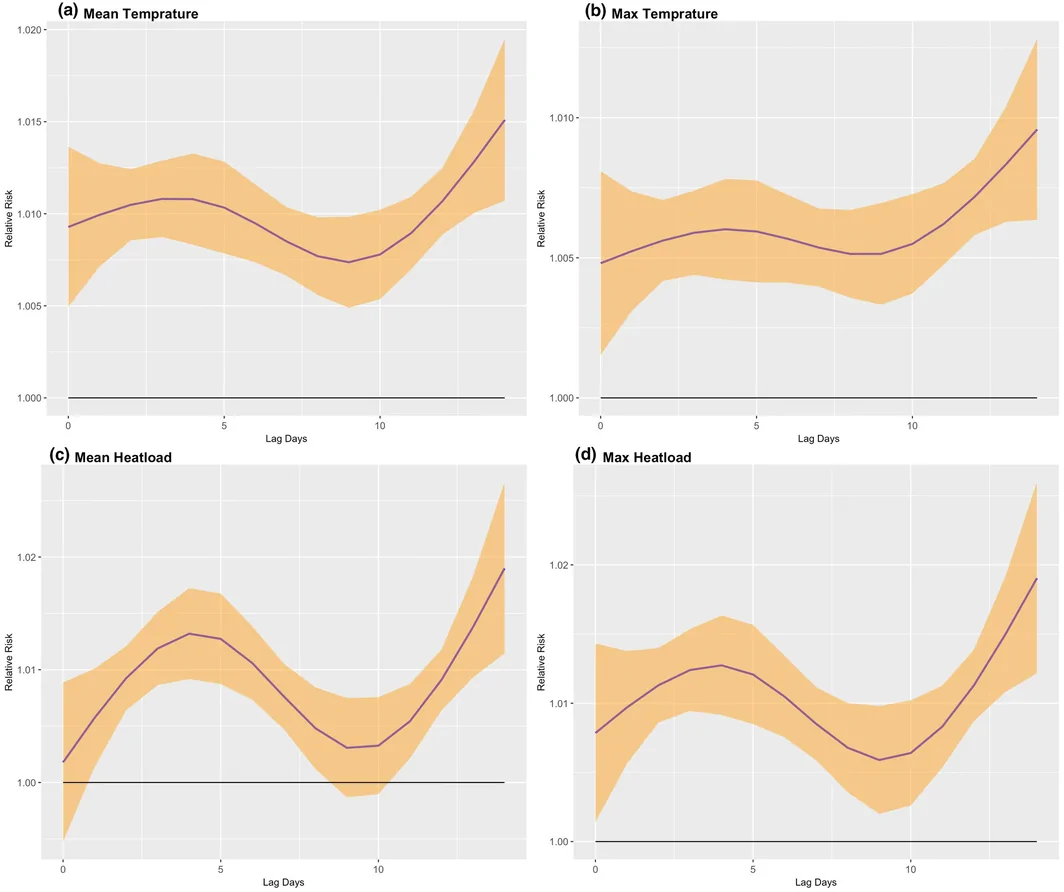
Impact of temperature and heat‐load increase on the daily number of preterm births (<37th gestational week) ‐ individual lag days effect. (a) Mean temperature. (b) Maximal temperature. (c) Mean heat load. (d) Maximal heat load. Comment: Associations between acute exposure to a 1°C increase in mean and maximal temperature and mean and maximal heat load on individual days and the risk for preterm birth (gestational week <37). For a 1°C increase in mean daily temperature, the relative risk (RR) for the first day after exposure was 1.009 (95% confidence interval [CI]: 1.007 to 1.012), for the eighth day following exposure the RR = 1.007 (95% CI: 1.005–1.009) and for the 14 day after exposure, the RR was 1.015 (95% CI: 1.010–1.019). Similarly, for a 1°C increase in maximal heat load, the RR for the first day after exposure was 1.005 (95% CI: 1.001–1.010), for the eighth day following exposure the RR = 1.004 (95% CI: 1.001–1.008) and for the 14th day after exposure, the RR was 1.018 (95% CI: 1.011–1.026). The cross‐basis for lag effects was modeled using four degrees of freedom to capture potential non‐linear and delayed associations.

Heat load (also referred to as the discomfort index) data were obtained from the IMS, based on measurements from meteorological monitoring stations, where it is defined as the arithmetic mean of the dry bulb (ambient) and wet bulb temperatures, reflecting the combined effects of temperature and humidity. This metric captures physiological heat burden more effectively than ambient temperature alone, as human thermoregulation relies on evaporative cooling, which is impaired under conditions of high humidity.^24^, ^25^ Consequently, identical ambient temperatures may result in different levels of heat stress depending on humidity, making composite measures such as heat load more representative of perceived and physiological thermal stress. This index has also been used in prior epidemiologic studies to evaluate the impact of environmental heat exposure on human health outcomes.^26^, ^27^

### 2.3 Definition of seasonality

Synoptic seasonality is a concept that identifies distinct natural seasons based on the persistence and dominance of specific synoptic systems in a given region.^20^ In the Eastern Mediterranean, for example, synoptic seasonality is characterized by the prevalence of systems like the Persian trough in summer, Cyprus low in winter, Red Sea^28^, ^29^ trough in autumn, and Sharav lows in spring, with observable changes in their frequency marking the beginning and end of each season.

Seasonality was defined according to the Israeli daily synoptic scale^20^ meteorology system, as follows: spring (March 31–May 30), summer (May 31–September 22), autumn (September 23–December 6), and winter (December 7–March 30).

We combined the databases according to the relevant date and district and created a time‐series of births, preterm births and early preterm births, with daily meteorological indices.

### 2.4 Statistical analysis

All statistical analyses were performed using R version 4.5.1.^30^ We calculated the mean, minimum, and maximum temperature, heat load, and number of preterm births by season and birth year across the different districts. The mean daily number of preterm births was compared between the different seasons and between the different birth years, as a whole and in each specific district, using the analysis of variance (ANOVA).

We constructed models to investigate the effect of four indices (mean daily temperature, maximum daily temperature, mean heat load, and maximal heat load) on four primary outcomes: the daily number of daily early preterm (<32nd gestational week) births (out of total daily births), preterm births (between 32–37 gestational weeks), total preterm births (<37 gestational week) and births that resulted in very low birth weight (<1500 g) newborns.

In evaluating the impacts of temperature on daily preterm births, we employed statistical modeling to examine both the immediate and delayed effects. Specifically, we utilized generalized linear models (GLMs) with a Poisson distribution, to account for the number of preterm and very low birth weight births.

Additionally, to capture the delayed influences of temperature, we implemented non‐linear distributed lag models (DLNMs).^21^, ^22^ DLNMs offer a bidimensional framework, allowing us to examine both delayed and non‐linear relationships between the four indices and the different outcomes. Our analysis encompassed a 14‐day lag period between exposure and outcome. Here, lag‐0 signifies the immediate effect, while lag‐14 represents the association with temperature experienced 14 days before the current date. This method ensures a detailed examination of the complex interplay between temperature fluctuations and the daily number of preterm births.

We modeled the lag constraint, that is, the restriction on how the temperature effect varies between the lagged days, using a natural cubic spline with four degrees of freedom. We also used natural cubic spline with four degrees of freedom to model the association between the daily temperature and the daily number of preterm births. The cross‐basis was constructed using a 14‐day lag period, with default quantile‐based knot placement as implemented in the dlnm package,^22^ and the exposure was modeled on its original scale without centering. The models were adjusted for the current season, the week's day, and the official Israeli holidays. We also added the trend as a predictor using a natural cubic spline with seven degrees of freedom per year.

### 2.5 Sensitivity analysis

We conducted several sensitivity analyses to ensure the robustness of our findings. To address potential model over‐flexibility, we constructed models with lag constraints using a natural cubic spline with two and three degrees of freedom. These models aimed to smooth potential fluctuations in the data while maintaining enough flexibility to capture significant trends. Additionally, to account for variations in the rate, rather than the absolute number of preterm births, we constructed models that incorporated the total number of daily births as an offset variable. Also, to address potential exposure misclassification arising from the use of a single central monitoring station to represent each district, we conducted a sensitivity analysis using a broader set of meteorological data. Specifically, we selected five stations, strategically scattered throughout the district within each district and re‐estimated the models using district‐level exposure defined by the mean temperature and heat load averaged across these stations. The DLNMs were then repeated using these alternative exposure metrics to assess the robustness of the main findings. Moreover, we compared seasonal temperature distributions and summary metrics between the 2010–2016 and 2017–2024 periods across all districts and seasons, to assess temporal stability in exposure patterns.

## 3 RESULTS

A total of 1 216 040 babies were born in Israel between 2010 and 2016, of which 89 544 (7.36%) were born before the 37th, and 11 154 (0.91%) were born before the 32nd gestational week.

The mean, minimum, and maximum temperature, heat load, and number of preterm births by season and birth year across the different districts are presented in Appendix S2 and Appendix S3, respectively. The mean daily number of preterm births was significantly higher in the summer (7.23) compared to the autumn (7.15), winter (6.9), and spring (6.3) (*P* < 0.001).

### 3.1 Daily number of total preterm births (<37 gestational weeks)

We found significant non‐linear associations between all four indices (the daily mean and maximum temperature, and the daily mean and maximal heat load) and the total daily number of preterm births (Figure 1). Specifically, for a 1°C increase in mean daily temperature, the relative risks (RR) for acute exposure on individual days were RR = 1.009 (95% confidence interval [CI]: 1.007–1.012) on the first day after exposure, RR = 1.007 (95% CI: 1.005–1.009) on the eighth day and RR = 1.015 (95% CI: 1.010–1.019) on the 14th day (Figure 1a).

Similarly, for a 1°C increase in maximal heat load, the RRs for acute exposure on individual days were RR = 1.005 (95% CI: 1.001–1.010) on the first day after exposure, RR = 1.004 (95% CI: 1.001 to 1.008) on the eighth day and RR = 1.018 (95% CI: 1.011–1.026) on the 14th day (Figure 1c).

Also, statistically significant cumulative effects were found between all four indices and the daily number of preterm births (RR = 1.160; 95% CI: 1.145–1.176 and RR = 1.139; 95% CI: 1.125–1.153 for 14‐days cumulative exposure to a 1°C increase in mean daily temperature and a 1°C increase in maximal heat load, respectively) (Figure 2).

**FIGURE 2:**
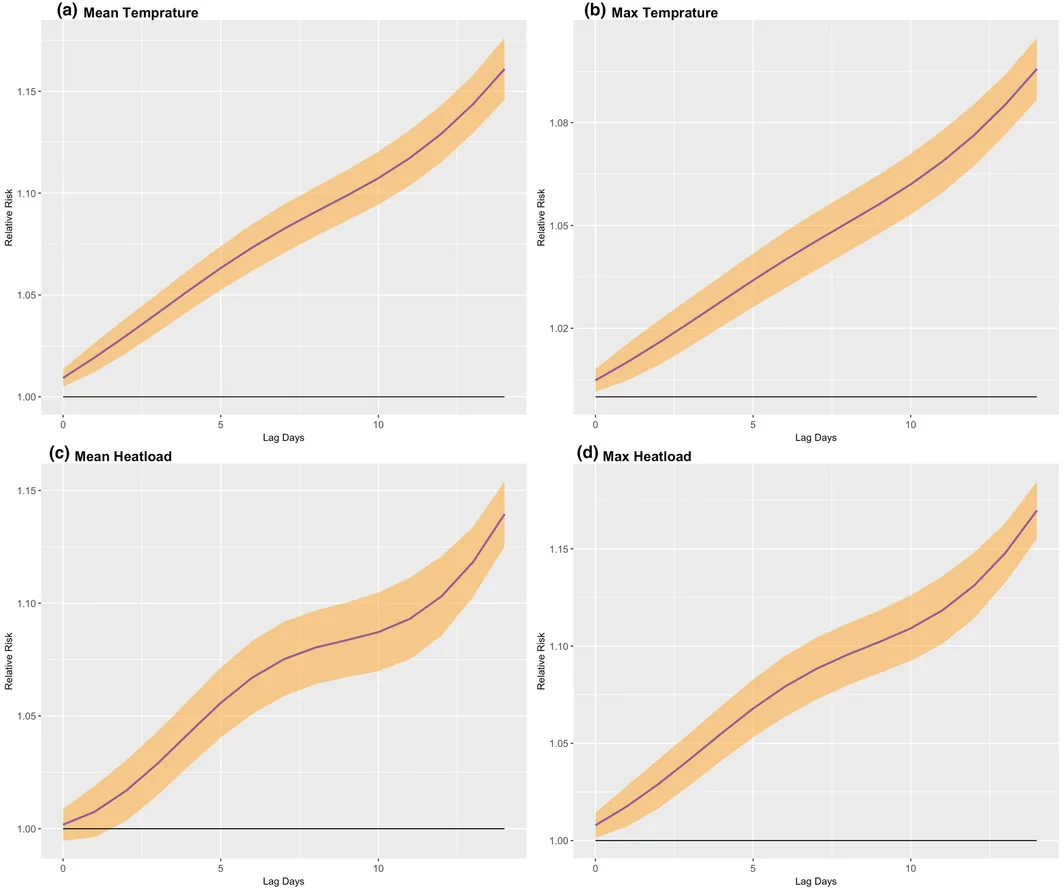
Cumulative impact of temperature and heat‐load increase on the daily number of preterm births (<37th gestational week). (a) Mean temperature. (b) Maximal temperature. (c) Mean heat load. (d) Maximal heat load. Comment: Cumulative effect of exposure to a 1°C increase in mean and maximal daily temperature, and a 1°C increase in mean and maximal heat load over 14 days, on the risk for preterm birth (gestational week <37). Statistically significant cumulative effects were found between all four indices and the number of daily preterm births (relative risk [RR] = 1.160; 95% confidence interval [CI]: 1.145–1.176 and RR = 1.139; 95% CI: 1.125–1.153 for 14‐days cumulative exposure to a 1°C increase in mean daily temperature and a 1°C increase in maximal heat load, respectively). The cross‐basis for lag effects was modeled using four degrees of freedom to capture potential non‐linear and delayed associations.

### 3.2 Daily number of early preterm (<32 gestational weeks) and very low birth weight births

Similar associations were found between all four indices and the daily number of early preterm births (Figure 3). For all indices, the maximal acute effect during the fifth day following exposure (RR = 1.019; 95% CI: 1.012–1.026 and RR = 1.021; 95% CI: 1.010–1.033 for a 1°C increase in mean daily temperature and maximal heat load, respectively). Also, statistically significant cumulative effects were found between all four indices and the number of daily early preterm births (RR = 1.194; 95% CI: 1.151–1.239 and RR = 1.161; 95% CI: 1.121–1.203 for 14 days cumulative exposure to a 1°C increase in mean daily temperature and maximal heat load, respectively) (Figure 4).

**FIGURE 3:**
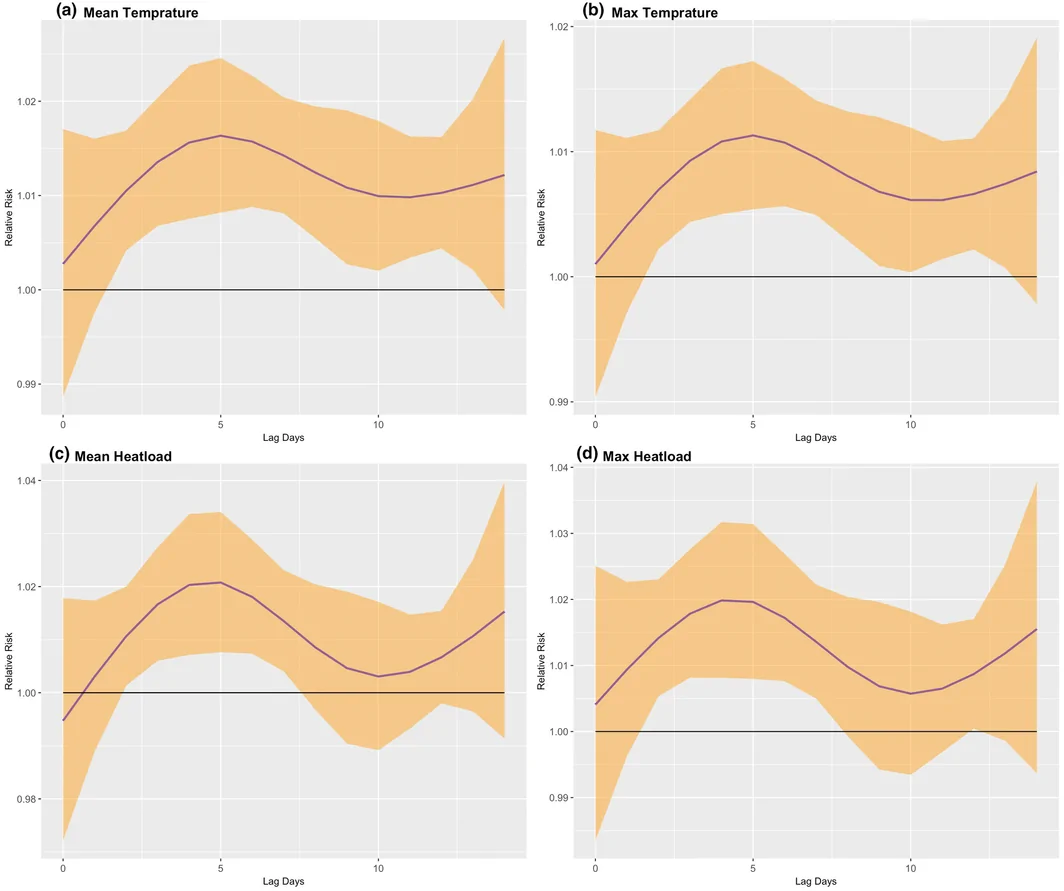
Impact of temperature and heat‐load increase on the daily number of early preterm births (<32nd gestational week) ‐ individual lag days effect. (a) Mean temperature. (b) Maximal temperature. (c) Mean heat load. (d) Maximal heat load. Comment: Associations between acute exposure to a 1°C increase in mean and maximal daily temperature and mean and maximal heat load on individual days, and the risk for early preterm birth (gestational week <32). For all indices, the maximal effect occurred during the fifth day following exposure (relative risk [RR] = 1.019; 95% confidence interval [CI]: 1.012–1.026 and RR = 1.021; 95% CI: 1.010–1.033 for a 1°C increase in mean daily temperature and a 1°C increase in maximal heat load, respectively). The cross‐basis for lag effects was modeled using four degrees of freedom to capture potential non‐linear and delayed associations.

**FIGURE 4:**
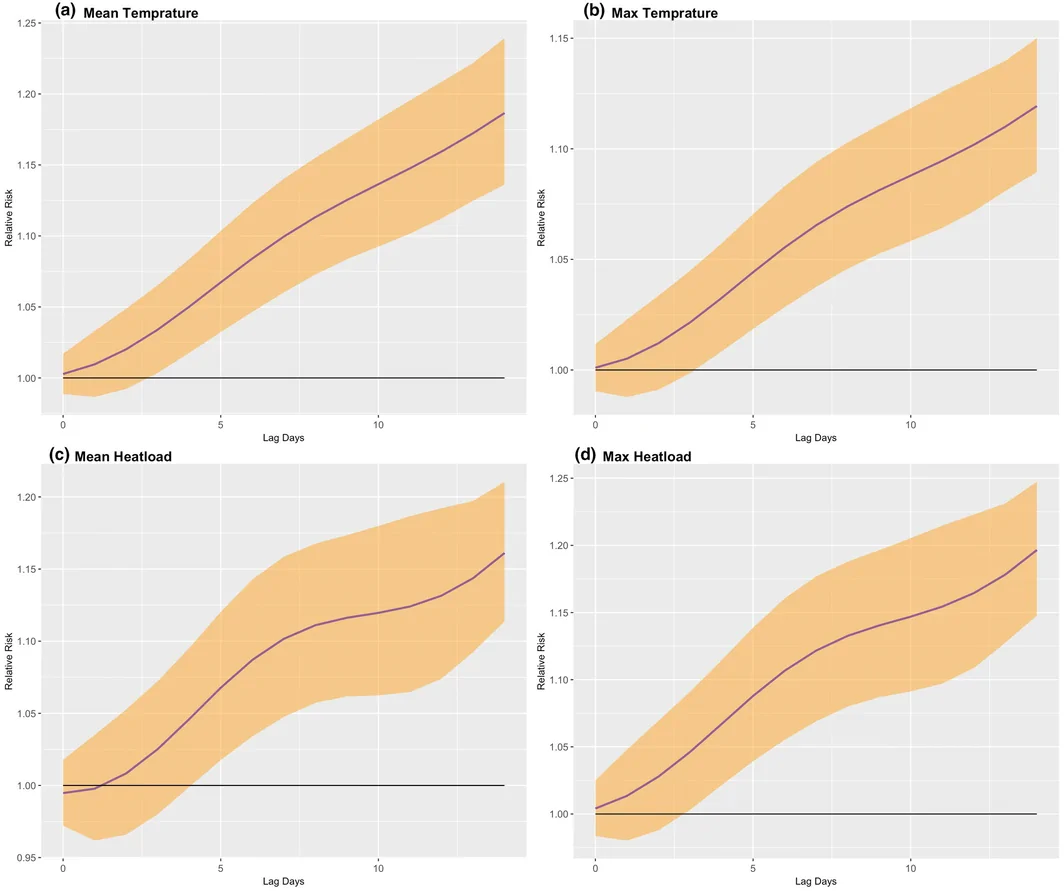
Cumulative impact of temperature and heat‐load increase on the daily number of early preterm births (<32nd gestational week). (a) Mean temperature. (b) Maximal temperature. (c) Mean heat load. (d) Maximal heat load. Comment: Cumulative effect of exposure to a 1°C increase in mean and maximal daily temperature, and a 1°C increase in mean and maximal heat load over 14 days, on the risk for preterm birth (gestational week <37). Statistically significant cumulative effects were found between all four indices and the number of daily early preterm births (relative risk [RR] = 1.194; 95% confidence interval [CI]: 1.151–1.239 and RR = 1.161; 95% CI: 1.121–1.203 for 14‐days cumulative exposure to a 1°C increase in mean daily temperature and a 1°C increase in maximal heat load, respectively). The cross‐basis for lag effects was modeled using four degrees of freedom to capture potential non‐linear and delayed associations.

We also observed both acute and cumulative associations for very low birth weight births (Appendices S4 and S5). The maximal acute effect was noted on the fifth day following exposure, with RR = 1.015 (95% CI: 1.008–1.022) for a 1°C increase in mean daily temperature and RR = 1.020 (95% CI: 1.007–1.034) for a 1°C increase in mean heat load (Appendix S4). Additionally, statistically significant cumulative effects were found across all four indices, with an increased daily number of very low birth weight births (RR = 1.186; 95% CI: 1.136–1.239 and RR = 1.161; 95% CI: 1.113–1.210 following 14 days of cumulative exposure to a 1°C increase in mean daily temperature and a 1°C increase in mean heat load) (Appendix S5).

### 3.3 Sensitivity analysis

Multiple sensitivity analyses were conducted. Models with two and three degrees of freedom, for both preterm and early preterm births, produced consistent results for both discrete and cumulative associations (Appendices S6–S13). Additionally, when analyzing the associations between the different indices and the daily rate of preterm births, rather than the total number of preterm births, the results remained similar (Appendices S14–S17).

In the sensitivity analysis using a five‐station exposure definition, results were consistent with the main findings. For preterm birth (<37 gestational weeks), a 1°C increase in mean temperature was associated with an increased risk on the first day after exposure (RR = 1.0119, 95% CI: 1.0072–1.0165), which persisted over subsequent days and peaked on day 14 after exposure (RR = 1.0159, 95% CI: 1.0129–1.0189), with a cumulative 14‐day effect of RR = 1.2019 (95% CI: 1.1828–1.2212). Similarly, a 1°C increase in mean heat load was associated with an elevated risk on the first day after exposure (RR = 1.0132, 95% CI: 1.0058–1.0206), reaching its maximum on day 14 (RR = 1.0190, 95% CI: 1.0145–1.0236), with a cumulative 14‐day effect of RR = 1.2480 (95% CI: 1.2292–1.2671) (Appendices S18, S19).

In early preterm births (<32 gestational weeks), results also remained broadly consistent with the main findings. A 1°C increase in mean temperature was associated with a gradual increase in risk over the lag period, reaching a peak at day 6 after exposure (RR = 1.0219, 95% CI: 1.0144–1.0294), and remaining elevated thereafter, with a cumulative 14‐day effect of RR = 1.2352 (95% CI: 1.1807–1.2922). Similarly, a 1°C increase in mean heat load showed a comparable pattern, with the highest risk occurring at day 5 after exposure (RR = 1.0252, 95% CI: 1.0135–1.0370), and a cumulative 14‐day effect of RR = 1.2377 (95% CI: 1.1866–1.2911) (Appendices S20 and S21).

Seasonal minimum, maximum, and mean temperatures were highly consistent between the 2010–2016 and 2017–2024 periods across all districts (Appendix S22).

## 4 DISCUSSION

The present study found significant non‐linear associations between daily mean and maximum temperature, as well as daily mean and maximal heat load, and the total daily rate of preterm births. Statistically significant cumulative effects were also found between all four indices and the rate of preterm births. Similar associations were observed for early preterm and for very low birth weight births.

The observed delayed effect of temperature and heat load on preterm births prompts several potential explanations. First, it is well‐documented that an inflammatory process is a significant risk factor for preterm delivery,^31^ accounting for approximately 40% of cases.^9^ The 5‐day interval between exposure to increased temperatures and heat load and preterm births may coincide with the time required for the progression of inflammation.^32^ Thus, exposure to warm temperatures might serve as a trigger for this chain of events leading to preterm delivery. Additionally, elevated temperatures may be associated with changes in blood pressure^33^, ^34^ during specific exposure windows throughout pregnancy, potentially contributing to the observed delayed effect. A second, delayed phase was observed around 14 days for total preterm births. This peak remained significant across alternative model specifications with two and three degrees of freedom, suggesting it is unlikely to be a modeling artifact. Biologically, this delayed effect may reflect the time required for heat‐induced changes in uteroplacental blood flow, vascular function, or cumulative maternal stress to influence fetal development.^35^, ^36^

We selected four degrees of freedom for the lag structure to provide sufficient flexibility to capture potential non‐linear and delayed associations between heat exposure and preterm birth. While higher degrees of freedom could introduce some risk of overfitting or increased sensitivity to extreme values, sensitivity analyses with two and three degrees of freedom produced similar patterns, supporting the robustness of our findings. While multiple exposures, outcomes, and lag structures were examined, these analyses were conducted within a prespecified, a priori hypothesis‐driven framework. Given that exposures and outcomes are statistically correlated across models and lag structures, we did not apply formal multiple testing correction. Instead, interpretation is based on the consistency of effect estimates across outcomes, exposure definitions, and sensitivity analyses.

The results of the present study were similar to those of several other studies. Auger et al.^14^ analyzed data from Montreal, Canada, spanning nearly three decades, comprising 206 929 term and 12 390 preterm singleton live births from June to September. Results showed that while extreme heat episodes were associated with a 27% greater hazard of early‐term delivery compared to other days, there were no significant associations between maximum temperatures and preterm delivery. Kent et al.^15^ used a time‐stratified case‐crossover design to explore the correlation between heat wave days and preterm births in Alabama, USA, from 1990 to 2010. Findings reveal that heat waves, defined as at least two consecutive days with mean daily temperatures above the 98th percentile, are linked to a substantial 32.4% increase in preterm birth (PTB) risk. Additionally, heat waves defined with mean daily temperatures above the 90th percentile demonstrate a 3.7% higher risk of preterm births. Ha et al.^19^ investigated the correlation between ambient temperature exposure and early deliveries using medical records from 223 375 singleton deliveries across 12 US sites.^19^ Using Poisson regression and generalized estimating equations, the authors found that exposure to hot or cold temperatures during specific periods of pregnancy increased the risk of early preterm, late preterm, and early term deliveries compared to mild temperatures. For instance, exposure to hot or cold temperatures during weeks 1–7 elevated the risk of early preterm deliveries by 11% and 20%, respectively. Similarly, whole‐pregnancy hot exposures increased delivery risk by 6%–21% at specific weeks. Additionally, in a case‐crossover analysis, a 5°F increase in temperature during the week preceding delivery was associated with 12%–16% higher risk of early delivery during warm seasons and 4%–5% lower risk during cold seasons. Ye et al.^16^ investigated associations between heat exposure, greenness, and PTB using data from births in Sydney, New South Wales, Australia, between 2000 and 2020. Exposures included greenness measured using normalized difference vegetation index (NDVI) and tree cover, along with daily extreme heat and nighttime extreme heat. Results showed that exposure to daily extreme heat and nighttime extreme heat during the third trimester was associated with increased risks of PTB, with adjusted odds ratios of 1.61 (95% CI: 1.55–1.67) and 1.51 (95% CI: 1.46–1.56), respectively. Disparities in associations were observed, with lower odds of PTB among pregnant individuals residing in greener areas. Basu et al.^17^ examined the link between heat and humidity, measured by apparent temperature, and preterm delivery. Analyzing nearly 60 000 births across 16 California counties from 1999 to 2006, they found a significant association between high ambient temperature and preterm birth across all maternal demographic groups, independent of air pollutants. Specifically, a 10°F (5.6°C) increase in weekly average apparent temperature was associated with an 8.6% rise in preterm delivery. Cox et al.^18^ analyzed data from 807 835 singleton deliveries occurring between January 1998 and July 2011. Employing a quasi‐Poisson model combined with distributed lag non‐linear models, the researchers investigated the effects of temperature on preterm birth, considering delayed and non‐linear effects while accounting for daily pregnancies at risk and their gestational age distribution. Results indicated that moderate heat, occurring up to one day before delivery, increased the risk of preterm birth by 8.5%–9.6%, with corresponding estimates for extreme heat at 15.6%–14.5%. Cold temperatures had minimal cumulative effects on preterm birth, with a slight increase in risk associated with moderate cold up to three days before delivery.

In contrast to previous studies, the present study stands out in several key aspects. First, except for one study, it is the only one to utilize a sophisticated distributed lag non‐linear model (DLNM), enabling the association of non‐linear relationships with lagged effects up to 14 days before delivery. This advanced modeling approach provided insights that allowed the current study to recognize the fifth day before delivery as the most vulnerable period, shedding light on a specific timeframe crucial for preventive interventions. Moreover, the study incorporated heat load as a measure of temperature exposure, which accounts for both temperature and humidity, providing a more holistic assessment of environmental influences on preterm birth. Lastly, the current study utilized comprehensive data from Israel, encompassing a diverse range of climatic conditions throughout the year, not limited to the summer months.

The findings of the present study have significant clinical implications for prenatal care and public health interventions. First, by highlighting the increased risk of preterm birth associated with heat load and high temperatures, healthcare providers can suggest specific guidance to pregnant women, advising them to avoid prolonged exposure to extreme heat. Second, recognizing the vulnerability of the fifth day before delivery, health providers can instruct pregnant women to prioritize hydration and take preventive measures during the period following extreme weather events to mitigate the risk of early labor. These targeted interventions have the potential to reduce the incidence of preterm births and improve maternal and fetal health outcomes.

The research implications of this study extend to advancing our understanding of the complex relationship between environmental factors and preterm births. By utilizing advanced DLNMs, this study provides valuable insights into the temporal dynamics of temperature and heat load exposure on gestational outcomes. Furthermore, future research can build upon these findings by exploring additional factors that may interact with environmental exposures to influence preterm birth risk, such as maternal health status, socioeconomic factors, and air pollution levels. Also, investigating the effectiveness of targeted interventions aimed at reducing heat‐related preterm births can help develop evidence‐based strategies to mitigate the adverse effects of environmental stressors during pregnancy. Overall, this study sets a foundation for further investigation into the mechanisms underlying preterm birth and informs the development of preventive measures to promote maternal and infant health.

One of the key strengths of our study lies in the utilization of DLNMs,^21^, ^22^ which offer a sophisticated approach to unraveling the intricate relationship between temperature exposure and preterm birth risk. DLNMs enable the investigation of non‐linear associations and capture lagged effects over time, revealing critical windows of vulnerability, such as the fifth day before delivery. This flexibility allows for a comprehensive understanding of the temporal dynamics between environmental factors and gestational outcomes, enhancing the identification of preventive intervention opportunities.

Furthermore, our study benefits from the diverse climatic conditions present in Israel, influenced by its varied topography.^20^ Israel experiences pronounced seasonal variations, notably in its desert regions, with distinct weather patterns characterized by a cool and rainy winter season extending from October to April, followed by a dry and hot summer season lasting from May to September. This diversity in weather conditions offers a unique opportunity to explore the relationship between temperature fluctuations and preterm deliveries within varied environmental contexts.

Moreover, the present study has the advantage of including a diverse range of demographics from both urban and rural areas within Israel.^37^, ^38^ This diversity contributes to the complexity of the sociodemographic environment and enhances the external validity of our results. By encompassing individuals from various ethnic, religious, and socioeconomic backgrounds, our study captures a more comprehensive picture of the association between temperature variations and preterm births, ensuring applicability across different demographic groups. Additionally, our study's basis on the entire Israeli population adds robustness and generalizability to the findings, as it captures a diverse range of demographic and environmental factors that may influence preterm birth risk.

Furthermore, the robustness of our findings is strengthened by the adjustments made in our models. Notably, we incorporated adjustments for the current season, the day of the week, and official Israeli holidays, which helps to account for potential confounding factors related to temporal variation and societal patterns. Additionally, the inclusion of a trend predictor enhances the accuracy of our analyses by capturing long‐term temporal trends in preterm birth rates. These adjustments further contribute to the reliability and validity of our results. Our study had several limitations. We could not differentiate between spontaneous preterm birth and medically indicated preterm birth. These phenotypes may have distinct underlying mechanisms and differential susceptibility to heat‐related exposures and combining them may introduce bias into the observed associations. Another limitation is the lack of adjustment for additional environmental and socioeconomic factors, including socioeconomic status, residential greenness, and air pollution, all of which have been associated with preterm birth risk in previous studies.^39^, ^40^, ^41^ This may have introduced residual confounding into the observed associations. A possible limitation of this study is potential exposure misclassification arising from the use of ambient temperature and heat load from a single monitoring station per region as proxies for individual‐level exposure. To address this, we conducted a sensitivity analysis using a five‐station average within each district to better capture spatial variability and achieved similar results.

Another limitation is the restricted study period (2010–2016), as more recent data were not available for linkage and validation. However, the distribution and seasonal structure of temperature in the study region have remained broadly stable over time, which reduces concern about major changes in exposure patterns.

## 5 CONCLUSION

In conclusion, our study revealed significant non‐linear associations between daily mean and maximal temperature, and daily mean and maximal heat load, and both the total daily rate of preterm births and early preterm births. Notably, the fifth day before delivery emerged as a particularly vulnerable period. Furthermore, significant cumulative effects were observed across all four indices and the rate of preterm births. These findings highlight the significant impact of high temperature and heat load on the risk of preterm birth. This emphasizes the need for specific interventions and preventive measures to reduce both short‐ and long‐term morbidity associated with prematurity.

## AUTHOR CONTRIBUTIONS

The corresponding author confirms that all bylined authors, including Bernard Barzilay, Abu Kishk Ibrahim, Haim Bibi, Dan Waisman, and Sharon Daniel, have substantially contributed to the concept and design, analysis and interpretation of data, and drafted the manuscript or revised it critically for important intellectual content. All authors approved the submitted version of the manuscript.

## CONFLICT OF INTEREST STATEMENT

The authors declare no conflict of interest.

## DECLARATION

During the preparation of this work, the author(s) did not use any generative AI tools or AI‐assisted technologies. The content was reviewed and edited as needed by the author(s).

## Supporting information


**Appendix S1.** Meteorological measuring stations and locations by district.
**Appendix S2.** Minimal, maximal, and mean temperature and heat load, and the rate of preterm births, across different seasons and residential areas.
**Appendix S3.** Minimal, maximal, and mean temperature and heat load, and the rate of preterm births along the study years, between the different residential areas.
**Appendix S4.** Impact of temperature and heat‐load increase on the daily number of very low birth weight (<1500 g) births ‐ individual lag days effect. (a) Mean temperature. (b) Maximum temperature. (c) Mean heat load. (d) Maximal heat load.
**Appendix S5.** Cumulative impact of temperature and heat‐load increase on the daily number of very low birth weight (<1500 g) births. (a) Mean temperature. (b) Maximum temperature. (c) Mean heat load. (d) Maximal heat load.
**Appendix S6.** Impact of temperature and heat‐load increase on the daily number of preterm births (<37th gestational week) ‐ individual lag days effect, models with two degrees of freedom. (a) Mean temperature. (b) Maximum temperature. (c) Mean heat load. (d) Maximal heat load.
**Appendix S7.** Cumulative impact of temperature and heat‐load increase on the daily number of preterm births (<37th gestational week), models with two degrees of freedom. (a) Mean temperature. (b) Maximum temperature. (c) Mean heat load. (d) Maximal heat load.
**Appendix S8.** Impact of temperature and heat‐load increase on the daily number of early preterm births (<32nd gestational week) ‐ individual lag days effect, models with two degrees of freedom. (a) Mean temperature. (b) Maximum temperature. (c) Mean heat load. (d) Maximal heat load.
**Appendix S9.** Cumulative impact of temperature and heat‐load increase on the daily number of early preterm births (<32nd gestational week), models with two degrees of freedom. (a) Mean temperature. (b) Maximum temperature. (c) Mean heat load. (d) Maximal heat load.
**Appendix S10.** Impact of temperature and heat‐load increase on the daily number of preterm births (<37th gestational week) ‐ individual lag days effect, models with three degrees of freedom. (a) Mean temperature. (b) Maximum temperature. (c) Mean heat load. (d) Maximal heat load.
**Appendix S11.** Cumulative impact of temperature and heat‐load increase on the daily number of preterm births (<37th gestational week), models with three degrees of freedom. (a) Mean temperature. (b) Maximum temperature. (c) Mean heat load. (d) Maximal heat load.
**Appendix S12.** Impact of temperature and heat‐load increase on the daily number of early preterm births (<32nd gestational week) ‐ individual lag days effect, models with three degrees of freedom. (a) Mean temperature. (b) Maximum temperature. (c) Mean heat load. (d) Maximal heat load.
**Appendix S13.** Cumulative impact of temperature and heat‐load increase on the daily number of early preterm births (<32nd gestational week), models with three degrees of freedom. (a) Mean temperature. (b) Maximum temperature. (c) Mean heat load. (d) Maximal heat load.
**Appendix S14.** Impact of temperature and heat‐load increase on the daily rate of preterm births (<37th gestational week) ‐ individual lag days effect. (a) Mean temperature. (b) Maximum temperature. (c) Mean heat load. (d) Maximum heat load.
**Appendix S15.** Cumulative impact of temperature and heat‐load increase on the daily rate of preterm births (<37th gestational week). (a) Mean temperature. (b) Maximum temperature. (c) Mean heat load. (d) Maximal heat load.
**Appendix S16.** Impact of temperature and heat‐load increase on the daily rate of early preterm births (<32nd gestational week) ‐ individual lag days effect. (a) Mean temperature. (b) Maximum temperature. (c) Mean heat load. (d) Maximal heat load.
**Appendix S17.** Cumulative impact of temperature and heat‐load increase on the daily rate of early preterm births (<32nd gestational week). (a) Mean temperature. (b) Maximum temperature. (c) Mean heat load. (d) Maximal heat load.
**Appendix S18.** Sensitivity analysis – acute impact of mean temperature and mean heat load across districts (5‐station average) on the daily rate of preterm births (<37 weeks' gestation) – individual lag day effects. (a) Mean temperature. (b) Mean heat load.
**Appendix S19.** Sensitivity analysis – cumulative impact of mean temperature and mean heat load across districts (5‐station average) on the daily rate of preterm births (<37 weeks' gestation) – individual lag day effects. (a) Mean temperature. (b) Mean heat load.
**Appendix S20.** Sensitivity analysis – acute impact of mean temperature and mean heat load across districts (5‐station average) on the daily rate of early preterm births (<32 weeks' gestation) – individual lag day effects. (a) Mean temperature. (b) Mean heat load.
**Appendix S21.** Sensitivity analysis – cumulative impact of mean temperature and mean heat load across districts (5‐station average) on the daily rate of early preterm births (<32 weeks' gestation) – individual lag day effects. (a) Mean temperature. (b) Mean heat load.
**Appendix S22.** Comparison of seasonal temperature metrics by region between 2010–2016 and 2017–2024.

## Data Availability

The data that support the findings of this study are not publicly available due to strict privacy and confidentiality regulations governed by the Israeli Ministry of Health. Researchers may request access to the data directly from the Israeli Ministry of Health, subject to approval and compliance with all applicable data security and ethical requirements.
