# Supplementary material for: Temperature and heat‐load exposure effects on preterm births: Insights from a population‐based study using distributed lag non‐linear models
Source: Int J Gynaecol Obstet. 2026 Jun 5;175(2):930–40. doi: 10.1002/ijgo.71112 (PMC13674827; doi:10.1002/ijgo.71112)
Supplement: Supplementary file 1 [file IJGO-175-930-s001.docx]

**Supplemental Materials:

Temperature and Heat-Load Exposure Effects on Preterm Births: Insights from a Population-Based Study Using Distributed Lag Non-linear Models**

Bernard Barzilay MD^1,2^ , Abu Kishk Ibrahim MD^2,3^, Haim Bibi MD^1,4^, Dan Waisman MD*^5,6^, Daniel Sharon MD,MPH,PhD *^7,8,9^

^1^Mayanei Hayeshua Medical Center, Bnei Brak. Israel. ^2^Sackler Faculty of Medicine, Tel Aviv University, Tel Aviv, Israel. ^3^Pediatric Intensive Care Unit, Shamir Medical Center (Assaf Harofeh), Zerifin, Israel.^4^Adelson school of Medicine, Ariel University, Ariel, Israel. ^5^Carmel Medical Center, Haifa, Israel. ^6^The Rappaport School of Medicine, Technion, Israel Institute of Technology, Haifa, Israel. Faculty of Health Sciences, Departments of ^7^Public Health and ^8^Pediatrics, Ben-Gurion University of the Negev, Beer Sheva, Israel. ^9^Clalit Health Services (Southern District) Beer Sheva, Israel.

Table of Contents

[Appendix 1: Meteorological Measuring Stations and Locations by District	4](#_Toc227655211)

[Appendix 2: Minimal, Maximal, and Mean Temperature and Heat-load, and the Rate of Preterm Births, Across Different Seasons and Residential Areas	5](#_Toc227655212)

[Appendix 3: Minimal, Maximal, and Mean Temperature and Heat-load, and the Rate of Preterm Births along the study years, between the different residential areas	6](#_Toc227655213)

[Temperature. C. Mean Heatload. D. Max Heatload	7](#_Toc227655214)

[Appendix 5: Cumulative impact of Temperature and Heatload Increase on the daily number of very low birthweight (<1,500 gr) births. A. Mean Temperature. B. Max Temperature. C. Mean Heatload. D. Max Heatload	8](#_Toc227655215)

[Appendix 6: Impact of Temperature and Heatload Increase on the daily number of Preterm Births (<37th Gestational Week) - Individual Lag Days effect, models with 2 degrees of freedom. A. Mean Temperature. B. Max Temperature. C. Mean Heatload. D. Max Heatload	9](#_Toc227655216)

[Appendix 7: Cumulative impact of Temperature and Heatload Increase on the daily number of Preterm Births (<37th Gestational Week), models with 2 degrees of freedom. A. Mean Temperature. B. Max Temperature. C. Mean Heatload. D. Max Heatload	10](#_Toc227655217)

[Appendix 8: Impact of Temperature and Heatload Increase on the daily number of early-preterm Births (<32th Gestational Week) - Individual Lag Days effect, models with 2 degrees of freedom. A. Mean Temperature. B. Max Temperature. C. Mean Heatload. D. Max Heatload	11](#_Toc227655218)

[Appendix 9: Cumulative impact of Temperature and Heatload Increase on the daily number of early-preterm Births (<32th Gestational Week), models with 2 degrees of freedom. A. Mean Temperature. B. Max Temperature. C. Mean Heatload. D. Max Heatload	12](#_Toc227655219)

[Appendix 10: Impact of Temperature and Heatload Increase on the daily number of Preterm Births (<37th Gestational Week) - Individual Lag Days effect, models with 3 degrees of freedom. A. Mean Temperature. B. Max Temperature. C. Mean Heatload. D. Max Heatload	13](#_Toc227655220)

[Appendix 11: Cumulative impact of Temperature and Heatload Increase on the daily number of Preterm Births (<37th Gestational Week), models with 3 degrees of freedom. A. Mean Temperature. B. Max Temperature. C. Mean Heatload. D. Max Heatload	14](#_Toc227655221)

[Appendix 12: Impact of Temperature and Heatload Increase on the daily number of early-preterm Births (<32th Gestational Week) - Individual Lag Days effect, models with 3 degrees of freedom. A. Mean Temperature. B. Max Temperature. C. Mean Heatload. D. Max Heatload	15](#_Toc227655222)

[Appendix 13: Cumulative impact of Temperature and Heatload Increase on the daily number of early-preterm Births (<32th Gestational Week), models with 3 degrees of freedom. A. Mean Temperature. B. Max Temperature. C. Mean Heatload. D. Max Heatload	16](#_Toc227655223)

[Appendix 14: Impact of Temperature and Heatload Increase on the daily rate of Preterm Births (<37th Gestational Week) - Individual Lag Days effect. A. Mean Temperature. B. Max Temperature. C. Mean Heatload. D. Max Heatload	17](#_Toc227655224)

[Appendix 15: Cumulative impact of Temperature and Heatload Increase on the daily rate of Preterm Births (<37th Gestational Week). A. Mean Temperature. B. Max Temperature. C. Mean Heatload. D. Max Heatload	18](#_Toc227655225)

[Appendix 16: Impact of Temperature and Heatload Increase on the daily rate of early-preterm Births (<32th Gestational Week) - Individual Lag Days effect. A. Mean Temperature. B. Max Temperature. C. Mean Heatload. D. Max Heatload	19](#_Toc227655226)

[Appendix 17: Cumulative impact of Temperature and Heatload Increase on the daily rate of early-preterm Births (<32th Gestational Week). A. Mean Temperature. B. Max Temperature. C. Mean Heatload. D. Max Heatload	20](#_Toc227655227)

[Appendix 18: Sensitivity Analysis – Acute Impact of Mean Temperature and Mean Heat Load Across District (Five-Station Average) on the Daily Rate of Preterm Births (<37th Gestational Week) – Individual Lag Day Effects. A. Mean Temperature. B. Mean Heatload.	21](#_Toc227655228)

[Appendix 19: Sensitivity Analysis – Cumulative Impact of Mean Temperature and Mean Heat Load Across District (Five-Station Average) on the Daily Rate of Preterm Births (<37th Gestational Week) – Individual Lag Day Effects. A. Mean Temperature. B. Mean Heatload.	22](#_Toc227655229)

[Appendix 20: Sensitivity Analysis – Acute Impact of Mean Temperature and Mean Heat Load Across District (Five-Station Average) on the Daily Rate of Early Preterm Births (<32th Gestational Week) – Individual Lag Day Effects. A. Mean Temperature. B. Mean Heatload.	23](#_Toc227655230)

[Appendix 21: Sensitivity Analysis – Cumulative Impact of Mean Temperature and Mean Heat Load Across District (Five-Station Average) on the Daily Rate of Early Preterm Births (<32th Gestational Week) – Individual Lag Day Effects. A. Mean Temperature. B. Mean Heatload.	24](#_Toc227655231)

[Appendix 22: Comparison of Seasonal Temperature Metrics by Region Between 2010–2016 and 2017–2024.	25](#_Toc227655232)

## **Appendix 1**: Meteorological Measuring Stations and Locations by District

## **Appendix 2**: Minimal, Maximal, and Mean Temperature and Heat-load, and the Rate of Preterm Births, Across Different Seasons and Residential Areas

| Region | Season | Minimal Temperature | Maximal Temperature | Mean Temperature | Minimal Heat Load | Maximal Heat Load | Mean Heat Load | Preterm births <32 GW – daily mean | Preterm births <37 GW – daily mean |
| --- | --- | --- | --- | --- | --- | --- | --- | --- | --- |
| Jerusalem | Summer | 14 | 39.5 | 22.14 | 4.5 | 25.5 | 15.18 | 5.49 | 47.37 |
|  | Autumn | 3 | 31.2 | 13.04 | -1.3 | 18.2 | 8.57 | 5.41 | 46.69 |
|  | Winter | -1.8 | 35.2 | 14.52 | -2.7 | 22.1 | 10.21 | 5.36 | 46.62 |
|  | Spring | 5.5 | 39.7 | 24.18 | 10.6 | 25.1 | 18.39 | 5 | 40.67 |
| North | Summer | 15.8 | 39.4 | 22.43 | 5.3 | 27.2 | 18.29 | 5.67 | 41.23 |
|  | Autumn | 4.7 | 32.1 | 14.13 | -1.9 | 20.3 | 10.63 | 5.39 | 39.21 |
|  | Winter | -0.9 | 37.5 | 15.97 | -2.1 | 23.4 | 11.92 | 4.84 | 38.93 |
|  | Spring | 5.5 | 38.6 | 25.53 | 12.5 | 27.7 | 21.31 | 4.72 | 36.13 |
| Haifa | Summer | 17.2 | 37.8 | 22.69 | 7.8 | 28 | 19.1 | 3.39 | 25.03 |
|  | Autumn | 7.8 | 33.4 | 15.08 | -0.6 | 21 | 11.42 | 3.35 | 24.23 |
|  | Winter | 2.3 | 37.5 | 16.98 | 0.4 | 23.8 | 13.17 | 2.86 | 23.42 |
|  | Spring | 8 | 34.7 | 25.83 | 13.3 | 27.7 | 22.28 | 2.57 | 21.07 |
| Tel Aviv | Summer | 16.6 | 41.8 | 24.51 | 6.9 | 27.9 | 19.81 | 11.17 | 96.57 |
|  | Autumn | 2.1 | 37.1 | 16.16 | 0.5 | 21.9 | 12.59 | 11.6 | 98.19 |
|  | Winter | 1.1 | 38.7 | 17.69 | -1.8 | 25.3 | 14.05 | 10.56 | 95.61 |
|  | Spring | 7 | 37.8 | 27.69 | 12.6 | 28.6 | 22.99 | 10.48 | 87.97 |
| South | Summer | 14.4 | 42.9 | 24.76 | 4 | 27.8 | 18.49 | 6.23 | 42.84 |
|  | Autumn | 1.7 | 37.2 | 15.62 | 0.1 | 23.4 | 11.35 | 6.12 | 43.01 |
|  | Winter | 0.5 | 41 | 16.65 | -0.7 | 24.2 | 12.58 | 5.65 | 41.35 |
|  | Spring | 6 | 41.6 | 26.94 | 11 | 28.7 | 21.6 | 5.97 | 36.43 |

## **Appendix 3:** Minimal, Maximal, and Mean Temperature and Heat-load, and the Rate of Preterm Births along the study years, between the different residential areas

| Region | Year | Minimal Temperature | Maximal Temperature | Mean Temperature | Minimal Heat Load | Maximal Heat Load | Mean Heat Load | Number of preterm births <32 GW | Number of preterm births <37 GW |
| --- | --- | --- | --- | --- | --- | --- | --- | --- | --- |
| Jerusalem | 2010 | 1.7 | 36.3 | 18.46 | -1.3 | 23.5 | 12.85 | 294 | 2467 |
|  | 2011 | 2.9 | 39.5 | 17.99 | -2.7 | 25.1 | 12.71 | 278 | 2459 |
|  | 2012 | 0.7 | 36.4 | 18.19 | 0.2 | 23.5 | 12.89 | 286 | 2411 |
|  | 2013 | -0.3 | 34.9 | 17.86 | -0.5 | 22.6 | 12.38 | 257 | 2444 |
|  | 2014 | 3 | 38.3 | 18.3 | 0.2 | 25 | 12.86 | 288 | 2437 |
|  | 2015 | -1.8 | 36.2 | 17.02 | 1.3 | 23.9 | 12.31 | 279 | 2462 |
|  | 2016 | 0 | 39.7 | 19.69 | -0.1 | 25.5 | 13.59 | 271 | 2066 |
| North | 2010 | 2.5 | 38.7 | 19.58 | -1.9 | 25.4 | 15.08 | 281 | 2125 |
|  | 2011 | 3.8 | 39.4 | 19.04 | -2.1 | 27.2 | 14.84 | 272 | 2122 |
|  | 2012 | 2.5 | 37.1 | 19.37 | 1.5 | 25.7 | 15.33 | 239 | 2003 |
|  | 2013 | 0.8 | 35 | 18.99 | 0.7 | 24.9 | 15.07 | 271 | 1942 |
|  | 2014 | 3.3 | 35.5 | 18.94 | 1.5 | 26.3 | 15.33 | 260 | 1970 |
|  | 2015 | -0.9 | 36.7 | 18.17 | 3.3 | 25.8 | 14.88 | 277 | 2088 |
|  | 2016 | 0.9 | 38.6 | 20.36 | 1.2 | 27.7 | 15.95 | 296 | 2074 |
| Haifa | 2010 | 4.2 | 36.3 | 20.34 | -0.6 | 25.1 | 16.05 | 167 | 1238 |
|  | 2011 | 6 | 37.8 | 19.83 | 0.4 | 28 | 15.99 | 178 | 1342 |
|  | 2012 | 4.8 | 37.6 | 19.97 | 2.6 | 25.9 | 16.25 | 167 | 1275 |
|  | 2013 | 3.3 | 33.7 | 19.83 | 3 | 25.7 | 15.96 | 153 | 1143 |
|  | 2014 | 6.9 | 32.6 | 19.5 | 2.4 | 26.5 | 16.35 | 151 | 1232 |
|  | 2015 | 2.3 | 35 | 18.84 | 4 | 25.1 | 15.71 | 145 | 1224 |
|  | 2016 | 3 | 37.5 | 20.68 | 2.6 | 27.7 | 16.6 | 163 | 1196 |
| Tel Aviv | 2010 | 3.2 | 39.8 | 21.3 | -1.8 | 26.3 | 16.89 | 574 | 5096 |
|  | 2011 | 4.6 | 41.8 | 20.89 | 1 | 28.6 | 16.77 | 571 | 5069 |
|  | 2012 | 3.5 | 40.2 | 21.01 | 1.9 | 26.3 | 16.88 | 617 | 4744 |
|  | 2013 | 1.4 | 37.4 | 21.1 | 1.2 | 26.3 | 16.78 | 525 | 4865 |
|  | 2014 | 3.3 | 37.7 | 21.16 | 1.6 | 27.6 | 17.37 | 615 | 5206 |
|  | 2015 | 1.3 | 38.4 | 20.76 | 3.7 | 26.7 | 16.87 | 584 | 5001 |
|  | 2016 | 1.1 | 38.7 | 22.03 | 2.9 | 27.9 | 17.72 | 512 | 4754 |
| South | 2010 | 5 | 42.3 | 20.7 | 0.1 | 25.8 | 15.39 | 295 | 2157 |
|  | 2011 | 4.5 | 42.9 | 20.32 | -0.7 | 27.8 | 15.41 | 300 | 2140 |
|  | 2012 | 2.8 | 39.9 | 20.33 | 1 | 25.3 | 15.52 | 340 | 2197 |
|  | 2013 | 2.4 | 40.9 | 20.32 | 1.9 | 24.5 | 15.16 | 324 | 2174 |
|  | 2014 | 2.8 | 40.5 | 20.58 | 0.9 | 27.2 | 15.94 | 271 | 2121 |
|  | 2015 | 1.7 | 40.5 | 20.12 | 2.4 | 26.4 | 15.45 | 342 | 2158 |
|  | 2016 | 0.5 | 42.2 | 22.48 | 3.1 | 28.7 | 16.89 | 311 | 2142 |

**Appendix 4**: Impact of Temperature and Heatload Increase on the daily number of very low birthweight (<1,500 gr) births - Individual Lag Days effect. A. Mean Temperature. B. Max

## Temperature. C. Mean Heatload. D. Max Heatload

**Appendix 5**: Cumulative impact of Temperature and Heatload Increase on the daily number of very low birthweight (<1,500 gr) births. A. Mean Temperature. B. Max Temperature. C. Mean Heatload. D. Max Heatload

**Appendix 6**: Impact of Temperature and Heatload Increase on the daily number of Preterm Births (<37th Gestational Week) - Individual Lag Days effect, models with 2 degrees of freedom. A. Mean Temperature. B. Max Temperature. C. Mean Heatload. D. Max Heatload

**Appendix 7**: Cumulative impact of Temperature and Heatload Increase on the daily number of Preterm Births (<37th Gestational Week), models with 2 degrees of freedom. A. Mean Temperature. B. Max Temperature. C. Mean Heatload. D. Max Heatload

**Appendix 8**: Impact of Temperature and Heatload Increase on the daily number of early-preterm Births (<32th Gestational Week) - Individual Lag Days effect, models with 2 degrees of freedom. A. Mean Temperature. B. Max Temperature. C. Mean Heatload. D. Max Heatload

**Appendix 9**: Cumulative impact of Temperature and Heatload Increase on the daily number of early-preterm Births (<32th Gestational Week), models with 2 degrees of freedom. A. Mean Temperature. B. Max Temperature. C. Mean Heatload. D. Max Heatload

**Appendix 10**: Impact of Temperature and Heatload Increase on the daily number of Preterm Births (<37th Gestational Week) - Individual Lag Days effect, models with 3 degrees of freedom. A. Mean Temperature. B. Max Temperature. C. Mean Heatload. D. Max Heatload

**Appendix 11:** Cumulative impact of Temperature and Heatload Increase on the daily number of Preterm Births (<37th Gestational Week), models with 3 degrees of freedom. A. Mean Temperature. B. Max Temperature. C. Mean Heatload. D. Max Heatload

**Appendix 12**: Impact of Temperature and Heatload Increase on the daily number of early-preterm Births (<32th Gestational Week) - Individual Lag Days effect, models with 3 degrees of freedom. A. Mean Temperature. B. Max Temperature. C. Mean Heatload. D. Max Heatload

**Appendix 13:** Cumulative impact of Temperature and Heatload Increase on the daily number of early-preterm Births (<32th Gestational Week), models with 3 degrees of freedom. A. Mean Temperature. B. Max Temperature. C. Mean Heatload. D. Max Heatload

**Appendix 14**: Impact of Temperature and Heatload Increase on the daily rate of Preterm Births (<37th Gestational Week) - Individual Lag Days effect. A. Mean Temperature. B. Max Temperature. C. Mean Heatload. D. Max Heatload

**Appendix 15**: Cumulative impact of Temperature and Heatload Increase on the daily rate of Preterm Births (<37th Gestational Week). A. Mean Temperature. B. Max Temperature. C. Mean Heatload. D. Max Heatload

**Appendix 16**: Impact of Temperature and Heatload Increase on the daily rate of early-preterm Births (<32th Gestational Week) - Individual Lag Days effect. A. Mean Temperature. B. Max Temperature. C. Mean Heatload. D. Max Heatload

**Appendix 17**: Cumulative impact of Temperature and Heatload Increase on the daily rate of early-preterm Births (<32th Gestational Week). A. Mean Temperature. B. Max Temperature. C. Mean Heatload. D. Max Heatload

## **Appendix 18**: Sensitivity Analysis – Acute Impact of Mean Temperature and Mean Heat Load Across District (Five-Station Average) on the Daily Rate of Preterm Births (<37th Gestational Week) – Individual Lag Day Effects. A. Mean Temperature. B. Mean Heatload.

## **Appendix 19**: Sensitivity Analysis – Cumulative Impact of Mean Temperature and Mean Heat Load Across District (Five-Station Average) on the Daily Rate of Preterm Births (<37th Gestational Week) – Individual Lag Day Effects. A. Mean Temperature. B. Mean Heatload.

## **Appendix 20**: Sensitivity Analysis – Acute Impact of Mean Temperature and Mean Heat Load Across District (Five-Station Average) on the Daily Rate of Early Preterm Births (<32th Gestational Week) – Individual Lag Day Effects. A. Mean Temperature. B. Mean Heatload.

## **Appendix 21**: Sensitivity Analysis – Cumulative Impact of Mean Temperature and Mean Heat Load Across District (Five-Station Average) on the Daily Rate of Early Preterm Births (<32th Gestational Week) – Individual Lag Day Effects. A. Mean Temperature. B. Mean Heatload.

| **Region** | **Season** | **Min (2010–2016)** | **Min (2017–2024)** | **Max (2010–2016)** | **Max (2017–2024)** | **Mean (2010–2016)** | **Mean (2017–2024)** |
| --- | --- | --- | --- | --- | --- | --- | --- |
| **Jerusalem** | Summer | 19.9 | 20.3 | 30.1 | 30.7 | 25.0 | 25.5 |
|  | Autumn | 15.5 | 15.7 | 23.7 | 23.9 | 19.6 | 19.8 |
|  | Winter | 8.6 | 8.4 | 15.6 | 15.2 | 12.1 | 11.8 |
|  | Spring | 14.7 | 15.0 | 24.5 | 25.2 | 19.6 | 20.1 |
| **North** | Summer | 21.2 | 21.6 | 30.2 | 30.2 | 25.7 | 25.9 |
|  | Autumn | 16.7 | 17.2 | 25.6 | 25.5 | 21.1 | 21.4 |
|  | Winter | 9.5 | 9.7 | 17.2 | 16.9 | 13.4 | 13.3 |
|  | Spring | 15.1 | 15.4 | 25.2 | 25.4 | 20.1 | 20.4 |
| **Haifa** | Summer | 22.5 | 22.7 | 28.7 | 29.4 | 25.6 | 26.1 |
|  | Autumn | 17.7 | 18.2 | 24.7 | 25.5 | 21.2 | 21.9 |
|  | Winter | 11.0 | 11.0 | 17.6 | 17.9 | 14.3 | 14.4 |
|  | Spring | 16.0 | 16.2 | 24.3 | 24.9 | 20.1 | 20.6 |
| **Tel-Aviv** | Summer | 22.7 | 22.6 | 31.8 | 31.8 | 27.2 | 27.2 |
|  | Autumn | 16.2 | 16.5 | 27.6 | 27.6 | 21.9 | 22.0 |
|  | Winter | 9.7 | 9.5 | 20.7 | 20.3 | 15.2 | 14.9 |
|  | Spring | 15.5 | 15.4 | 26.9 | 27.2 | 21.2 | 21.3 |
| **South** | Summer | 21.2 | 21.2 | 34.5 | 34.6 | 27.9 | 27.9 |
|  | Autumn | 15.2 | 15.2 | 28.2 | 28.5 | 21.7 | 21.8 |
|  | Winter | 8.8 | 8.3 | 20.4 | 20.1 | 14.6 | 14.2 |
|  | Spring | 14.9 | 14.8 | 29.4 | 29.9 | 22.2 | 22.4 |

## **Appendix 22:** Comparison of Seasonal Temperature Metrics by Region Between 2010–2016 and 2017–2024.
